# Boosting the Piezoelectric Response and Interfacial Compatibility in Flexible Piezoelectric Composites via DET-Doping BT Nanoparticles

**DOI:** 10.3390/polym16060743

**Published:** 2024-03-08

**Authors:** Liming Liu, Hongjian Zhang, Shengyang Zhou, Changzhou Du, Ming Liu, Yong Zhang

**Affiliations:** State Key Laboratory of Silicate Materials for Architectures, Center for Smart Materials and Device Integration, School of Materials Science and Engineering, Wuhan University of Technology, Wuhan 430070, China; 317221@whut.edu.cn (L.L.); zhanghongjian@whut.edu.cn (H.Z.); 317341@whut.edu.cn (S.Z.); 268791@whut.edu.cn (C.D.); 317222@whut.edu.cn (M.L.)

**Keywords:** piezoelectric, energy harvesting, surface modification, ferroelectric

## Abstract

With the advent of the Internet of Things, self-powered wearable sensors have become increasingly prevalent in our daily lives. The utilization of piezoelectric composites to harness and sense surrounding mechanical vibrations has been extensively investigated during the last decades. However, the poor interface compatibility between ceramics nanofillers and polymers matrix, as well as low piezoelectric performance, still serves as a critical challenge. In this work, we employed Di(dioctylpyrophosphato) ethylene titanate (DET) as the coupling agent for modifying barium titanate (BTO) nanofillers. Compared to the BTO/PVDF counterpart, the DET-BTO/PVDF nanofibers exhibit an augmented content of piezoelectric *β* phase (~85.7%) and significantly enhanced stress transfer capability. The piezoelectric coefficient (d_33_) is up to ~40 pC/N, which is the highest value among reported BTO/PVDF composites. The piezoelectric energy harvesters (PEHs) present benign durability and attain a high instantaneous power density of 276.7 nW/cm^2^ at a matched load of 120 MΩ. Furthermore, the PEHs could sense various human activities, with the sensitivity as high as 0.817 V/N ranging from 0.05–0.1 N. This work proposes a new strategy to boosting the piezoelectric performance of PVDF-based composites via DET-doping ceramics nanoparticles, and in turn show significantly improved energy harvesting and sensing capability.

## 1. Introduction

The attention towards flexible pressure sensors has grown due to the rapid development of the Internet of Things (IoT) [1,2,3,4,5] and Artificial Intelligence (AI) [6,7,8,9]. Piezoelectric energy harvesters (PEHs) can harness the human body’s bioenergy, such as bending, vibration and oscillation, etc., as the power source for wireless and wearable electronics, which serves as a sustainable alternative to re-chargeable electrochemical batteries [10,11,12,13,14]. As the core of PEHs, piezoelectric materials determine the usage performance and application seniors. Piezoelectric composites, which take advantage of the high piezoelectric response of ceramics fillers and the intrinsic flexibility of polymer matrix simultaneously, are considered as a suitable choice for human motion energy harvesting and sensing [15,16,17,18,19,20].

PVDF is the typical matrix for piezoelectric composites, which exhibits five different crystalline phases: *α*, *β, γ*, *δ*, and *ε* [21,22,23,24]. Among them, the *β* phase, which is characterized by the all-trans (TTTT) conformation, exhibits the highest piezoelectric response [25,26]. However, *α* phase is the most stable one at room temperature. To induce the transition from *α* to *β* phase, several approaches, such as electrospinning, melt casting [27], annealing [28,29], polarization [30,31], and mechanical stretching [32,33] are proposed. Among them, electrospinning [34] has emerged as an effective method, as it utilizes mechanical stretching and high electric fields simultaneously to induce localized polarization. Furthermore, the interface between ceramics fillers and the PVDF matrix is another crucial factor for modulating the *β* phase content [35,36]. Due to the distinct characteristics of polymer and ceramics, they are not compatible with each other. Thus, the aggregation of ceramics fillers in polymer matrix is an intrinsically existed issue. As also demonstrated in previous studies, polar materials are beneficial for the formation of *β* phase in PVDF-based composites [37]. Besides, the existence of polar materials as the transition layer could boosting the evenly distribution of ceramics nanofillers in polymer matrix. Shi et al. prepared a flexible piezoelectric nanogenerator using Polymethylmethacrylate (PMMA)-coated BaTiO_3_ nanowires as the filler in a PVDF matrix. The incorporation of PMMA can boosting the weak interface and poor dispersion of BaTiO_3_ in PVDF [38]. As reported by Kim et al., Mxene is utilized as a nucleation agent to induce the formation of polar β phase, and in turn enhance the ferroelectric properties of PVDF [39].

Di(dioctylpyrophosphato) ethylene titanate (DET) is a commonly used coupling agent due to its multiple reactive groups, environmentally friendly nature, and cost-effectiveness [40,41]. Therefore, utilizing DET to modify BTO nanoparticles appears to be a promising approach. This modification helps to enhance the interface compatibility of ceramic-polymer composites and in turn facilitates the uniform dispersion of ceramic nanoparticles within the polymer matrix. Furthermore, the core–shell structured nanoparticles play a crucial role in boosting the polar *β* phase in PVDF due to the presence of functional groups of DET/BTO. In this study, DET-BTO/PVDF nanofibers were fabricated via the electrospinning method. The morphology and crystal structure of DET-BTO/PVDF nanofibers were systematically examined, as well as the dispersion of DET-BTO nanoparticles within the composite nanofibers. Various piezoelectric energy harvesters (PEHs) were prepared based on DET-BTO/PVDF fibers in conjunction with aluminum foil as the electrodes. The piezoelectric properties and durability of the resulting PEHs were investigated, in which the PEH containing 3 wt% DET-BTO/PVDF nanofibers exhibited the highest power density of 276.7 nW/cm^2^ under an external force of 18 N. Moreover, it exhibited remarkable stability during repeated pressing & releasing, without significant decrease even after 5000 cycles. The PEHs show benign sensitivity in detecting multiple human motions such as elbow flexion, finger tapping, pulse detection, and speech recognition.

## 2. Materials and Methods

### 2.1. Materials

BaTiO_3_, polyvinylidene fluoride (PVDF), Di(dioctylpyrophosphato) ethylene titanate (DET), N, N-dimethylformamide (C_3_H_7_NO, DMF), and acetone (CH_3_COCH_3_) were acquired from Aladdin, Shanghai, China. All chemicals were utilized as received without any additional treatment.

### 2.2. Modification of BTO Nanoparticles via DET

Firstly, 0.6 g of DET and 4 g of BTO were added sequentially to the deionized water (20 mL). The solution was then stirred at 60 °C for 3 h to guarantee the complete encapsulation of DET on BTO. The mixture was later centrifuged at a rate of 5000 rpm for 5 min to eliminate unreacted coupling agent. Finally, the obtained DET-BTO nanoparticles were washed with ethanol for three times and then dried at 60 °C for 12 h to remove remaining solvents.

### 2.3. Preparation of Electrospinning Fibers

A solvent for electrospinning was prepared by mixing DMF and acetone with a molar ratio of 3:2. The DET–BTO nanoparticles were then dissolved inside, followed by sonication (30 min) to ensure the uniform dispersion in DMF/acetone mixture. Subsequently, 17 wt% PVDF powder was introduced into the mixture and magnetic stirred at 60 °C for 3 h. This process facilitated the formation of hydrogen bonds, resulting in a stable and homogeneous blend solution of DET–BTO/PVDF for electrospinning. A series of electrospinning precursors were prepared using BTO/PVDF and DET–BTO/PVDF. The concentration of BTO and DET–BTO nanoparticles varied at 1 wt%, 3 wt%, and 5 wt% relative to the amount of PVDF. The solutions were injected into a 5 mL syringe with a flow rate of 1.5 mL/h. The syringe was connected to a flat-tipped needle with an inner diameter of 0.5 mm. The syringe nozzle was positioned 15 cm away from the grounded metal collector, and covered with aluminum foil to collect the resulting fibers. The drum speed was set as 200 rpm with an applied voltage of 18 kV maintained.

### 2.4. Characterizations

BTO, DET–BTO nanoparticles, BTO/PVDF and DET–BTO/PVDF were characterized using Cu Kα radiation X-ray diffraction (λ = 1.54 Å, operating at 40 kV and 20 mA, Rigaku Smartlab, Tokyo, Japan). Field emission scanning electron microscopy (FE-SEM, JSM-7610F, Rigaku, Tokyo, Japan) was used to characterize the morphology of PVDF-based composite fibers. The phase structure and surface functional groups of the prepared PVDF-based composite films were characterized using Fourier transform infrared spectroscopy (FT-IR, Nicolet6700, Bruker Spectrometer, Saarbrücken, Germany). The electrical signals generated by PEHs were collected by a digital oscilloscope (Keithley 6517B, Keithley, Solon, OH, USA).

## 3. Results and Discussion

**Figure 1:**
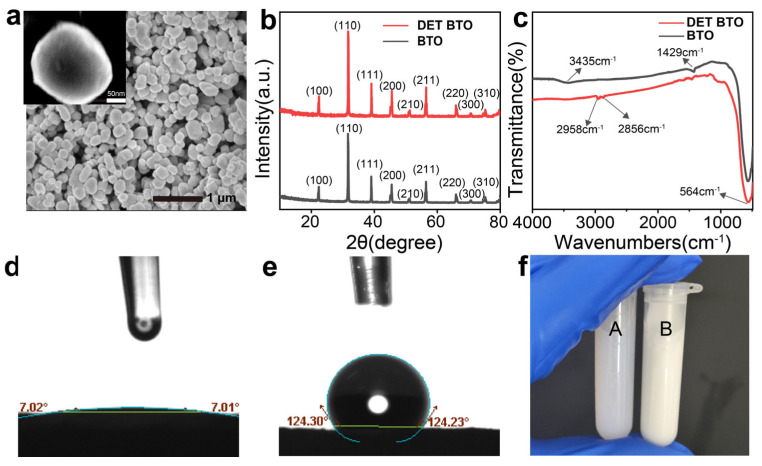
(**a**) SEM image of DET-BTO nanoparticles. (**b**,**c**) XRD patterns and FT-IR spectra of BTO and DET–BTO nanoparticles. (**d**,**e**) Contact angle of the hydrophilic BTO and hydrophobic DET-BTO. (**f**) BTO (**A**) and DET–BTO (**B**) in DMF (settled for 24 h after ultrasonic treatment for 30 min).

SEM (Figure 1a) images reveal a uniform particle size distribution (~100–300 nm) of DET-BTO nanoparticles. The surface modification did not change their crystalline phase, as confirmed with an XRD pattern (Figure 1b). The observed peak split at 45° is attributed to the presence of off-center Ti^4+^ ions resulting from the non-centrosymmetric tetragonal phase in BTO. To verify the presence of functional groups on the surface of DET–BTO nanoparticles, FT-IR spectra were recorded within 4000~400 cm^−1^ (Figure 1c). The Ti-O vibrational peak in BTO nanoparticles is observed at 564 cm^−1^. Concomitantly, characteristic -OH peaks are identifiable at 3435 cm^−1^ and 1429 cm^−1^. However, these -OH peaks are disappeared in the spectra of DET–BTO. Instead, more pronounced IR absorption bands are detected at 2958 cm^−1^ and 2856 cm^−1^, corresponding to the -CH_2_ stretching vibrations within the DET shells [42,43]. The observed variations imply that their surfaces have undergone grafting with long carbon chain coupling agents and modification coating. The contact angle of the DET–BTO exceeds 90° (Figure 1d,e), demonstrating its hydrophobic nature. In contrast, the unmodified BTO exhibits a contact angle of ~7°, indicating its hydrophilic characteristics. There is almost no change of wetting angle with time, proving the benign stability of the surface modification. The successful transformation of the BTO from hydrophilic to hydrophobic was achieved via DET modification. Ceramic particles that underwent ultrasonic dispersion for 0.5 h and settled in DMF for 24 h are shown in Figure 1f. The solution A with unmodified nanoparticles inside exhibited clarity, whereas the solution B containing DET–BTO nanoparticles displayed turbidity. This observation suggests the excellent dispersing performance of DET–BTO, as it resists agglomeration and precipitation.

**Figure 2:**
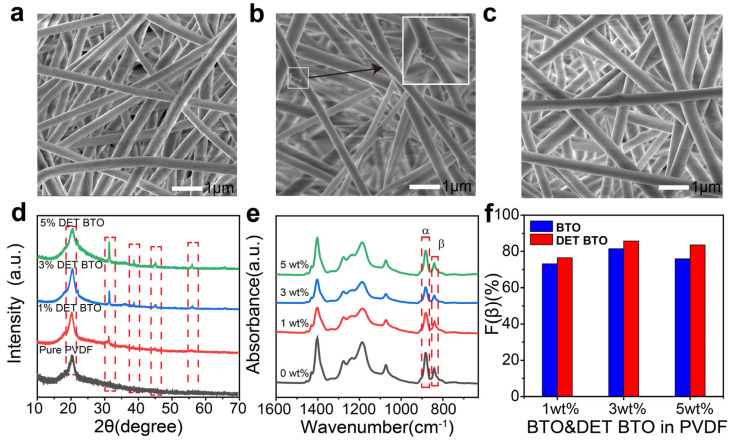
SEM images of the electrospinning fibers with (**a**) pure PVDF, (**b**) 3wt% BTO/PVDF, (**c**) 3wt% DET–BTO/PVDF. (**d**) XRD patterns of the electrospinning fibers with various DET–BTO mass fractions. (**e**) FT-IR spectra of the prepared electrospinning fibers with various DET–BTO mass fractions. (**f**) *β* phase fraction of the prepared electrospinning fibers with various BTO and DET–BTO mass fractions.

The morphology of pure PVDF, 3 wt% BTO/PVDF and 3 wt% DET–BTO/PVDF nanofibers was analyzed using SEM images (Figure 2a–c, Figures S1 and S2). Compared to BTO/PVDF nanofibers, the DET–BTO/PVDF nanofibers exhibit smoother and more continuous surface, as evident from the diameter distribution in Figure S2. BTO nanoparticles tend to agglomerate on or inside nanofibers, leading to a lack of uniformity in the resulting nanofibers. In contrast, DET–BTO nanoparticles were uniformly dispersed throughout the PVDF nanofibers. No obvious aggregation of DET–BTO nanoparticles in PVDF fiber could be observed. With DET modification on BTO nanoparticles, the diameter distribution of the fibers is concentrated around 350–500 nm (Figure S2f). The decrease in fiber diameter can be attributed to the hydrophobic nature of DET–BTO nanoparticles, which reduces their tendency to agglomerate and enables good interfacial compatibility with the hydrophobic PVDF. Additionally, the characteristic diffraction peak at 20.3° of XRD patterns (Figure 2d and Figure S3a) validates the presence of *β* phase. The piezoelectric response of PVDF is directly determined by the content of polar *β* phase. By utilizing FT-IR spectroscopy, we investigated the effect of DET–BTO and BTO concentrations on the *β* phase content of PVDF-based nanofibers (Figure 2e and Figure S3b). The characteristic absorption bands of *α* phase were observed at 763 cm^−1^ and 976 cm^−1^, while that of *β* phase were characterized at 840 cm^−1^ and 1275 cm^−1^. The *β* phase content in PVDF composites could be determined using the following equation [44,45,46].

$$F(\beta )=X_{\beta }/\left(X_{\alpha }+X_{\beta }\right)=A_{\beta }/\left(K_{\beta }/K_{\alpha }\right)A_{\alpha }+A_{\beta }$$

Here, *A_α_* and *A_β_* are the absorbance at 763 cm^−1^ and 840 cm^−1^, corresponding to the α phase and *β* phase of PVDF. *K_α_* and *K_β_* represent the absorption coefficients at the corresponding wavenumbers, which are 6.1 × 10^4^ and 7.7 × 10^4^ cm^2^ mol^−1^, respectively.

The FT-IR spectra (Figure 2e and Figure S3b) reveal small absorption peaks at 763 and 976 cm^−1^, indicating the presence of *α* phase. Additionally, larger peaks at 840 and 1275 cm^−1^ signify the dominance of *β* phase as the main crystalline structure. The *β* phase content of PVDF composite nanofibers is plotted in Figure 2f using the Lambert–Beer law [44,45,46]. During the electrospinning process, the strong electrostatic tension aligns the PVDF nanofibers in the polar direction, facilitating the accelerated formation of β phase. The *F(β)* values for DET–BTO/PVDF fibers with mass fractions of 1%, 3%, and 5% were measured as 76.5%, 85.7%, and 83.5% (Figure 2f), respectively. Initially, the *F(β)* values of the composite nanofibers were lower than that of the pure PVDF counterpart (84.6%), which can be attributed to the addition of BTO fillers in PVDF matrix [47]. The electrospinning precursor exhibits reduced conductivity, which impeded the movement of PVDF molecular chains to form *β* phase. With 3 wt% DET BTO fillers, the composite fiber exhibited the highest *β* phase content of 85.7%. Figure 2f clearly shows that the *β* phase of DET–BTO/PVDF were higher than that of BTO/PVDF, which well demonstrates the beneficial role of DET in enhancing the formation of *β* phase.

**Figure 3:**
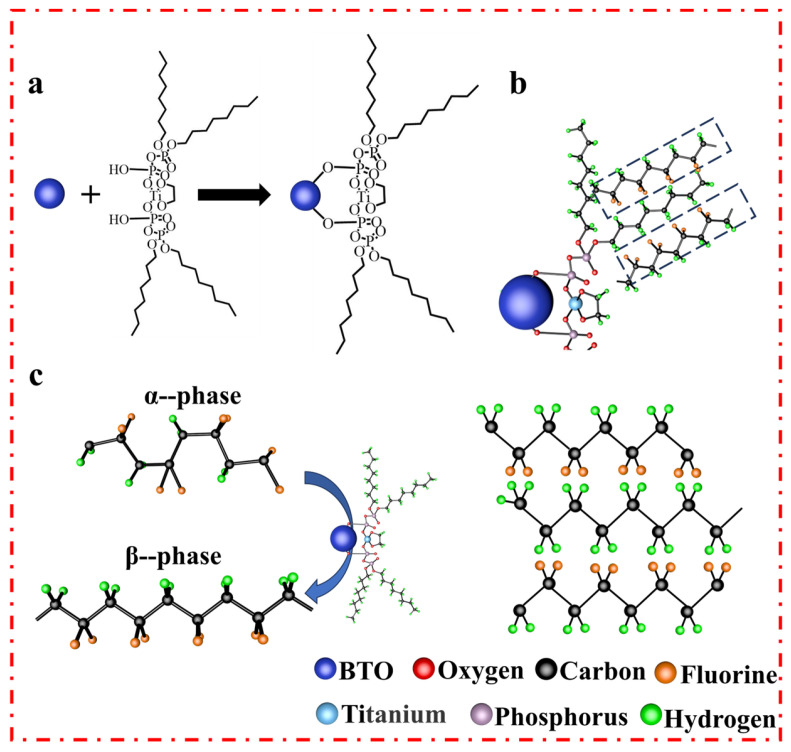
(**a**) Schematic of the interaction between BTO and DET. (**b**) The mechanism diagram of β phase formation in DET–BTO nanoparticles. (**c**) Schematic illustration of the transformation of α phase to *β* phase in PVDF-based films by the inclusion of DET–BTO.

Figure 3a illustrates the formation of a chemical bond between DET and BTO. This bond formation creates an interfacial layer between BTO nanofillers and PVDF matrix. The hydroxyl group within DET interacts with BTO nanoparticles through covalent bonding, resulting in an encapsulated layer on the surface of BTO [48]. This cross-linking reduces the surface activity of the nanoparticles. Moreover, DET–BTO nanoparticles disperse well in PVDF solution, facilitating their dispersion in PVDF-based nanofibers. Additionally, DET–BTO serves as a nucleating agent and adheres well to PVDF-based nanofibers, promoting the formation of oriented PVDF polymer chains. During the mixing process, as depicted in Figure 3b, the positively charged methylene groups within DET molecule interact with the negatively charged -CF_2_ along PVDF chains, leading to the formation of stable hydrogen bonds. These hydrogen bonds further facilitate the conversion of α phase to *β* phase in PVDF (Figure 3c). As a result, the compatibility and stability of BTO nanoparticles within PVDF matrix are enhanced, while preserving the stabilized PVDF chains. Overall, the formation of a chemical bond between DET and BTO nanoparticles, along with the interaction between DET and PVDF, contributes to the successful dispersion and stabilization of BTO nanoparticles in PVDF-based nanofibers. Furthermore, these interactions play a significant role in promoting the formation of desired *β* phase in PVDF, leading to improved piezoelectric response.

**Figure 4:**
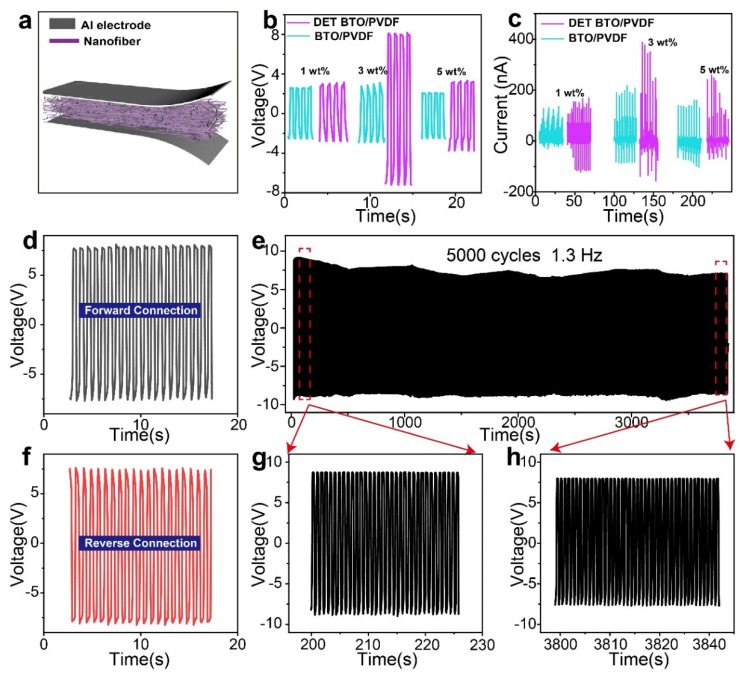
(**a**) Configuration of the PEHs. (**b**,**c**) The output voltage and current of the prepared fibers with various BTO and DET–BTO mass fractions under a fixed stress of 18 N. Real-time output with forward (**d**) and reversed (**f**) connection under a mechanical force of 16 N. (**e**) Mechanical stability test of 3 wt% DET–BTO/PVDF PEH with ~5000 times continuous impinging cycles at 18 N and 1.3 Hz. (**g**) Amplifying view of output voltage from 200 s to 225 s, (**h**) amplifying view of output voltage from 3798 s to 3831 s.

The samples were cut into square pieces (2 cm × 2 cm), with an aluminum foil as the electrodes for PEHs (Figure 4a). The energy harvesting capability of various PVDF-based composites with varying mass proportions (1, 3, and 5 wt%) of DET–BTO nanoparticles were characterized. The vertical pressure (18 N) generates a potential difference between two electrodes of PEHs. Upon the removal of the external force, the piezoelectric potential dissipates concurrently. Subsequently, a reverse current is generated followed by the flow of free electrons. As illustrated in Figure 4b,c and Figure S4, the output of PEHs is significantly influenced by the concentration of nanoparticles. The DET–BTO nanoparticles exhibited better dispersion within DET–BTO/PVDF, enhancing the compatibility between BTO and PVDF and thereby improving the piezoelectric properties. It is important to note that the DET–BTO/PVDF PEH presents higher output than that of BTO/PVDF PEH counterpart. The open-circuit voltage and short-circuit current of both unmodified and modified fibers reached their maximum values when the mass fraction of nanofillers was 3 wt%. Following DET modification, the output voltage and current increased from 3 V and 200 nA to 8.7 V and 400 nA, respectively. The performance enhancement is attributed to the DET layer, which improves the interface between BTO and PVDF and in turn higher stress-transfer capability and increased *β* phase content in PVDF. To affirm that the electrical signals are from the piezoelectric response rather than triboelectric or static charge generation, polarity tests were conducted. The induced charge flows in both directions, resulting in reversible voltage output (Figure 4d,f). The durability test (Figure 4e,g,h) demonstrating the benign mechanical durability, with the output experiences a drop of less than 10% even after 5000 cycles.

**Figure 5:**
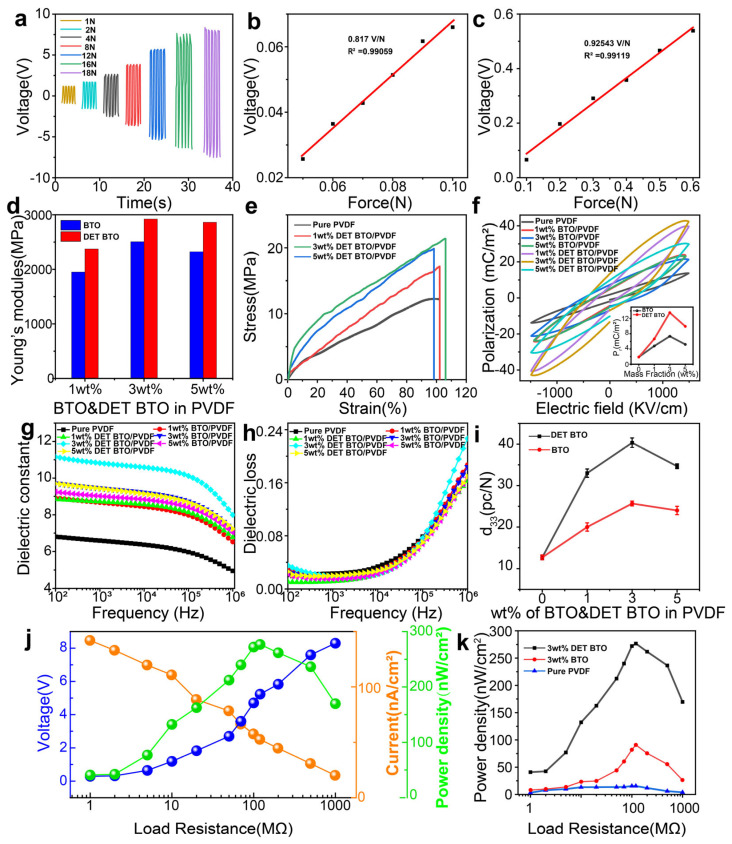
(**a**) Output voltage of the prepared fibers with 3 wt% DET–BTO under diverse external force. (**b**) The correlation between output voltage and pressure within 0.05–0.1 N. (**c**) The correlation between output voltage and pressure ranging from 0.1 N to 0.6 N. (**d**) Young’s modulus and € stress–strain curves of the prepared fibers with various DET–BTO and BTO mass fraction. (**e**) Stress-strain curves of the prepared electrospun fibers with various DET-BTO mass fraction.( (**f**) The hysteresis loops of the prepared fibers with various BTO and DET–BTO mass fractions at 1500 kV/cm. The inset image depicts the relationship between remnant polarization values and the mass fractions of BTO and DET–BTO at 1500 KV/cm. (**g**) The frequency dependence of dielectric constant. (**h**) Dielectric loss of various composite films. (**i**) d_33_ values of the prepared fibers with various BTO and DET–BTO mass fractions. (**j**) Voltage, current, and power density of 3 wt% DET–BTO/PVDF-based PEH at various load resistance. (**k**) The power density of pure PVDF, 3 wt% BTO, and DET–BTO/PVDF-based PEHs with various load resistance.

To illustrate the sensing properties of PEHs, we measured the output voltages of pure PVDF, BTO/PVDF and DET–BTO/PVDF under various force magnitudes. The output voltage exhibited a linear relationship with the force (Figure 5a and Figure S5). Additionally, the sensitivity was determined as 0.817 V/N ranging from 0.05–0.1 N, with a high linearity (R^2^) of 0.99059 (Figure 5b,c). Similarly, the sensitivity was calculated as 0.9253 V/N within 0.1–0.6 N, with a linearity (R^2^) of 0.99119. These findings demonstrate the exceptional sensing capability of the PEHs to subtle forces. The mechanical properties determine its ability to transmit stress from externally applied loads to flexible devices. Figure 5d,e and Figure S6 present stress–strain curves and Young’s modulus for various PVDF-based composites, which indicates a significant increase in tensile strength following the addition of BTO nanoparticles. For the polymer composites with same BTO content, the DET modified composites exhibit higher Young’s modulus compared to their unmodified counterparts. The maximum Young’s modulus is achieved at 3 wt% DET–BTO/PVDF of ~2920 MPa. The enhanced mechanical properties can be attributed to the exceptional adhesion and coupling facilitated by DET, resulting in strong intermolecular forces between the matrix and the DET shell on the BTO. After hot pressing at 110 °C, aluminum electrodes were coated on both sides of the composite film for in-poling process. The polarization process was conducted in two steps based on the reversed piezoelectric coefficient of PVDF and BTO. Firstly, the composite film was polarized at 40 kV/mm for 2 h. Then, it was polarized at 8 kV/mm for 0.5 h with the opposite polarization direction. The d_33_ values of various composite films were measured using a quasi-static d_33_ meter (Figure 5i and Figure S7), which increased from 12.6 pC/N for pure PVDF to 40.3 pC/N for 3 wt% DET–BTO/PVDF. Moreover, the d_33_ value of 3 wt% DET–BTO/PVDF was around 1.5 times higher than that of BTO/PVDF, indicating that DET modification can greatly enhance the piezoelectric performance. The numerous defects induced by the interface between PVDF and BTO could be alleviated via coating a DET layer on the surface of BTO nanofillers. As a result, the interfacial stress within the composite nanofibers between DET–BTO and PVDF exceeded that of BTO and PVDF. However, when the BTO concentration reached 5 wt%, the d_33_ value decreased to 34.6 pC/N. The decrease in d_33_ can be attributed to negative electromechanical coupling effect caused by BTO aggregation. The comparison between this work and previous studies is given in Table S1. The piezoelectric coefficient of 40.3 pC/N is higher than that in most previous studies. The ferroelectric and dielectric performance of various composite films were characterized and given in Figure 5f–h. The dielectric permittivity for all the composite films declines slightly with increasing frequency due to relaxation. The dielectric permittivity reaches the maximum at 3 wt% DET–BTO/PVDF composite film, with the dielectric loss is lower than 0.04 ranging from 0.1–1000 kHz. This is due to the proper inclusion of DET modification is beneficial for the interfacial polarization between nanofillers and polymer matrix. In general, the dielectric constant increases with the increase of ceramic fillers. For the same BTO content, the dielectric constant of DET–BTO/PVDF composites is higher than that of BTO/PVDF, which could be attributed to the fact that DET modification facilitates the uniform dispersion of ceramic nanoparticles within the polymer matrix and enhance the compatibility and interfacial polarization between the nanoparticles and the polymer matrix. Besides, the remanent polarization of DET–BTO/PVDF composites is around 2.5 times higher than that of BTO/PVDF counterpart, which induces the much-improved piezoelectric property. The energy harvesting capability of PEHs were characterized and shown in Figure 5j,k and Figure S8. The output voltage increases gradually with increasing load resistance and saturated at 8 V, while the current exhibits an opposite trend with increasing load resistance. Subsequently, the instantaneous power density of the PEHs was calculated by multiplying the voltage (V) and current (I) (Figure 5h). The maximum power density of 276.7 nW/cm^2^ was achieved at a matched load of 120 MΩ, which is 3.09 times higher than that of the unmodified PEH counterpart.

**Figure 6:**
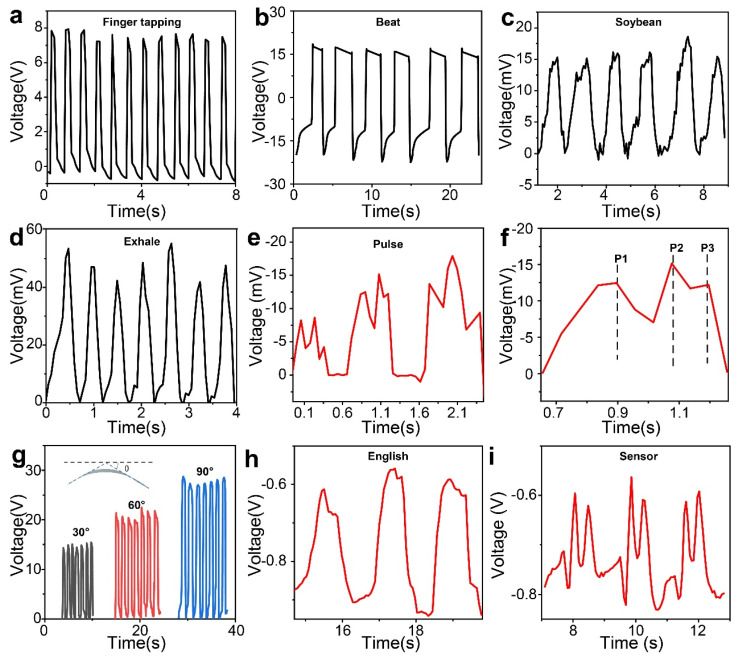
Output voltage of 3 wt% DET–BTO/PVDF-based PEH with (**a**) finger tapping, (**b**) beat, (**c**) soybean, and (**d**) exhale. (**e**) Real-time output voltage profiles of the pulse. (**f**) Enlarged views of the electrical signal consisting of three typical peaks (P1, P2, and P3). (**g**) Output voltage profile of finger bending at various angles. (**h**–**i**) Dynamic output profile for spontaneous voice recognition with different words. (**h**) English, (**i**) sensor.

The practical applications of PEHs were evaluated via sensing typical human actions such as finger pressing, and beating, for example (Figure 6a,b). These actions resulted in peak voltages of up to 28 V. Additionally, the PEHs could sense a very low force such as a soybean (Figure 6c), which highlighting the device’s benign sensitivity. Moreover, to validate the sensing capability in active physiological monitoring, we attached the device to a mask. When the wearer exhaled, the airflow expanded the mask and caused deformation on the attached sensor, thus generating an output signal waveform corresponding to the breathing pattern (Figure 6d). Furthermore, the device could detect the wrist pulse near subject’s wrist, which relies on the fact that small changes in arterial blood pressure result in slight distortions in the device, consequently producing corresponding signals (Figure 6e). Notably, an enlarged view of a single response peak (Figure 6f) revealed the presence of three distinct peaks representative of typical pulse waveforms (P1–P3) [49]. The above results well-demonstrate the rapid response time to subtle variations in pulse waveforms, which validates the great potential to monitor the physiological status of human cardiovascular system. Furthermore, to showcase the sensor’s ability to monitor limb movement, we affixed the fabricated sensor on the elbow to track the bending angle during movement (Figure 6g). Remarkably, the output voltage exhibited a linear relationship with the degree of elbow flexion, which can be attributed to the correlation between the bending angle of the elbow curl and the tensile deformation of the attached device. Additionally, after adhering the device to the neck, the pronunciation of “English” and “Sensor” induced vibrations in the neck that generated distinct piezoelectric signals, aligning with the characteristic pronunciation of each word (Figure 6h,i). Both “English” and “Sensor” are pronounced as disyllable with initial stress, which explains the voltage profile detected by the device: a strong peak followed by a weaker peak. These recurring stable signals demonstrate the successful replication of the sensing process. Taken together, these results indicate that the DET–BTO/PVDF devices exhibit outstanding capability in detecting various human movements.

## 4. Conclusions

In this study, DET was adopted for modifying BTO nanofillers. Compared to unmodified BTO, DET–BTO exhibits superior dispersion in PVDF solution. As was also demonstrated, the DET-doping BTO is beneficial for the interface compatibility between DET-BTO nanofillers and PVDF matrix, thereby improving stress transfer efficiency and promoting the formation of *β* phase in PVDF. The composite nanofibers were firstly prepared using electrospinning method, and then constructed as the PEHs. The addition of 3 wt% DET–BTO results in an instantaneous power density of 276.7 nW/cm^2^ at a matched load of 120 MΩ when subjected to an external force of 18 N, which is 3.09 times higher than the unmodified counterpart. Furthermore, the PEHs could sense various human activities, with the sensitivity as high as 0.817 V/N ranging from 0.05–0.1 N. Moreover, the fabricated PEHs were successfully utilized for pulse monitoring, identifying respiratory and voice recognition, etc. The PEHs demonstrated benign capability and potential in the fields of human–computer interaction and remote control for the Internet of Things (IoT).

## Data Availability

Data are contained within the article and Supplementary Materials.

## Supplementary Materials

The following supporting information can be downloaded at: https://www.mdpi.com/article/10.3390/polym16060743/s1, Figure S1: (a–d) SEM images of the prepared fibers with, (a) 1 wt% BTO/PVDF, (c) 5 wt% BTO/PVDF, (b) 1 wt% DET BTO/PVDF (d) 5 wt% DET BTO/PVDF; Figure S2: Fiber diameter distribution diagrams with, (a) pure PVDF, (b) 1wt% BTO/PVDF, (c) 3wt% BTO/PVDF, (d) 5wt% BTO/PVDF, (e) 1wt% DET BTO/PVDF, (f) 3wt% DET BTO/PVDF, (g) 5wt% DET BTO/PVDF; Figure S3: (a) X-ray diffraction (XRD) scattering pattern of the prepared electrospun fibers with various DET BTO mass fractions. (b) Fourier transform infrared (FTIR) spectra of the prepared electrospun fibers with various DET BTO mass fractions; Figure S4: The output voltage of the prepared fibers with pure PVDF under a fixed stress of 18N; Figure S5: Output voltage of the prepared fibers under diverse external force. (a) pure PVDF, (b) 1wt% BTO/PVDF, (c) 3 wt% BTO/PVDF, (d) 5 wt% BTO/PVDF, (e) 1wt% DET BTO/PVDF, (f) 5 wt% DET BTO/PVDF; Figure S6: Stress–strain curves of the prepared fibers with various BTO mass fractions; Figure S7: (a) Hysteresis loops of the prepared fibers with various BTO & DET BTO mass fractions at 1000 kV/cm, (b) The relationship between Pr values and the mass fractions of BTO & DET BTO at 1000 kV/cm; Figure S8: The d33 of (a) pure PVDF (b) 3 wt% BTO/PVDF (c) 3 wt% DET BTO/PVDF; Figure S9: Voltage, current and power density of PEHs with various load resistance. (a) pure PVDF, (b) 3 wt% BTO/PVDF; Table S1: Comparison between this work and previous studies; Video S1: D_33_ of 3wt% DET BTO/PVDF. Video S2: The Change Of Wetting Angle. References [43,50,51,52,53,54,55] are cited in the supplementary materials.
