# Supplementary material for: Boosting the Piezoelectric Response and Interfacial Compatibility in Flexible Piezoelectric Composites via DET-Doping BT Nanoparticles
Source: Polymers (Basel). 2024 Mar 8;16(6):743. doi: 10.3390/polym16060743 (PMC10974266; doi:10.3390/polym16060743)
Supplement: Supplementary file 1 [file polymers-16-00743-s001.zip › polymers-2886405-supplementary.pdf]

polymers MDPI

Supplementary Materials: Boosting the piezoelectric response and interfacial compatibility in flexible piezoelectric composites via DET doping BT nanoparticles

Liming Liu, Hongjian Zhang, Shengyang Zhou, Changzhou Du, Ming Liu, Yong Zhang

Highlights:

1. The DET-BTO/PVDF films exhibit dramatically improved piezoelectric coefficient of ~40 pC/N.
2. The improved interface compatibility is based on the interaction between positively charged methylene groups within DET molecule and negatively charged $-CF_2$ along PVDF chains, leading to the formation of stable hydrogen bonds.
3. The PEHs could sense various human activities, with the sensitivity as high as 0.817 V/N ranging from 0.05-0.1 N.

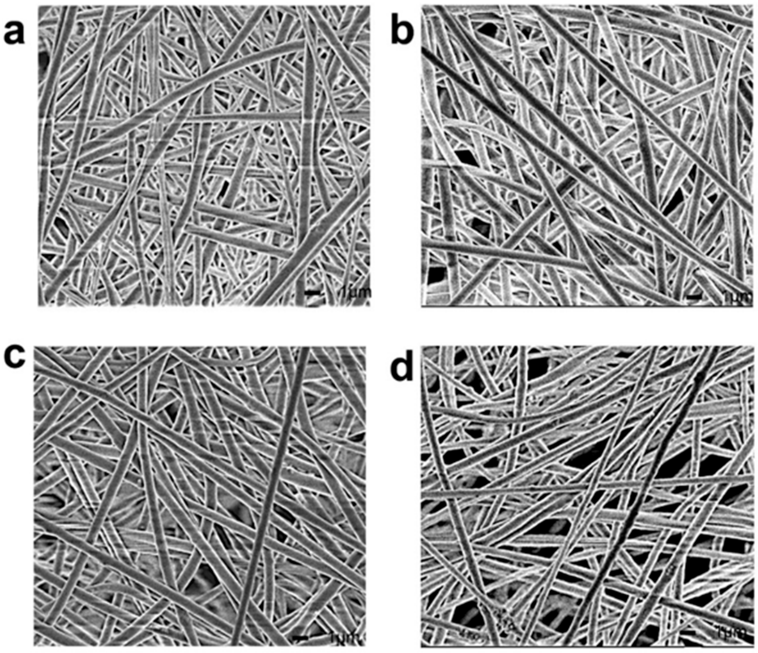

Figure S1. (a-d) SEM images of the prepared fibers with, (a) 1 wt% BTO/PVDF, (c) 5 wt% BTO/PVDF, (b) 1 wt% DET BTO/PVDF (d) 5 wt% DET BTO/PVDF.

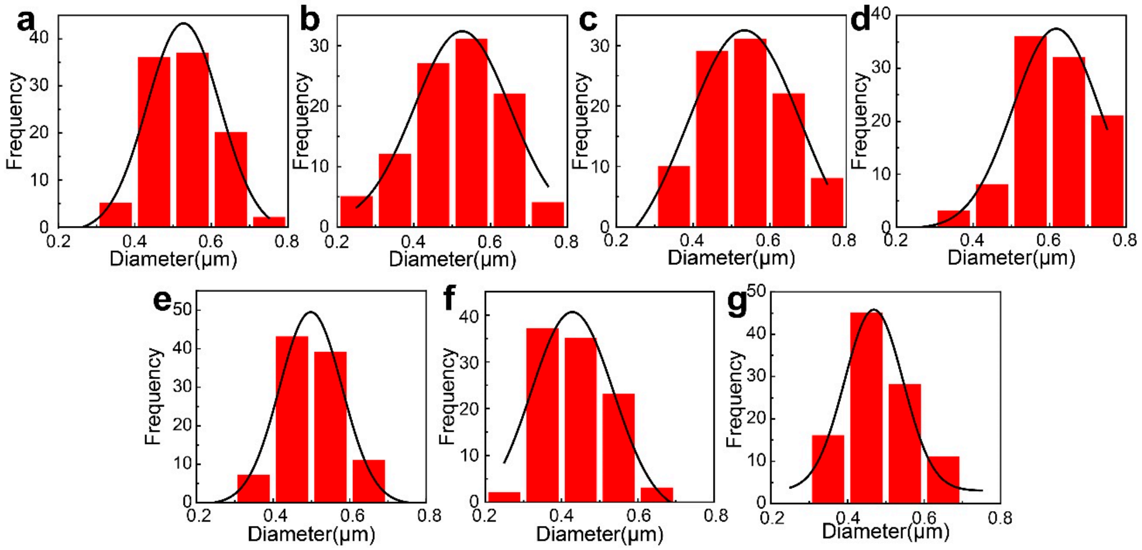

Figure S2. Fiber diameter distribution diagrams with, (a) pure PVDF, (b) 1wt% BTO/PVDF, (c) 3wt% BTO/PVDF, (d) 5wt% BTO/PVDF, (e) 1wt% DET BTO/PVDF, (f) 3wt% DET BTO/PVDF, (g) 5wt% DET BTO/PVDF.

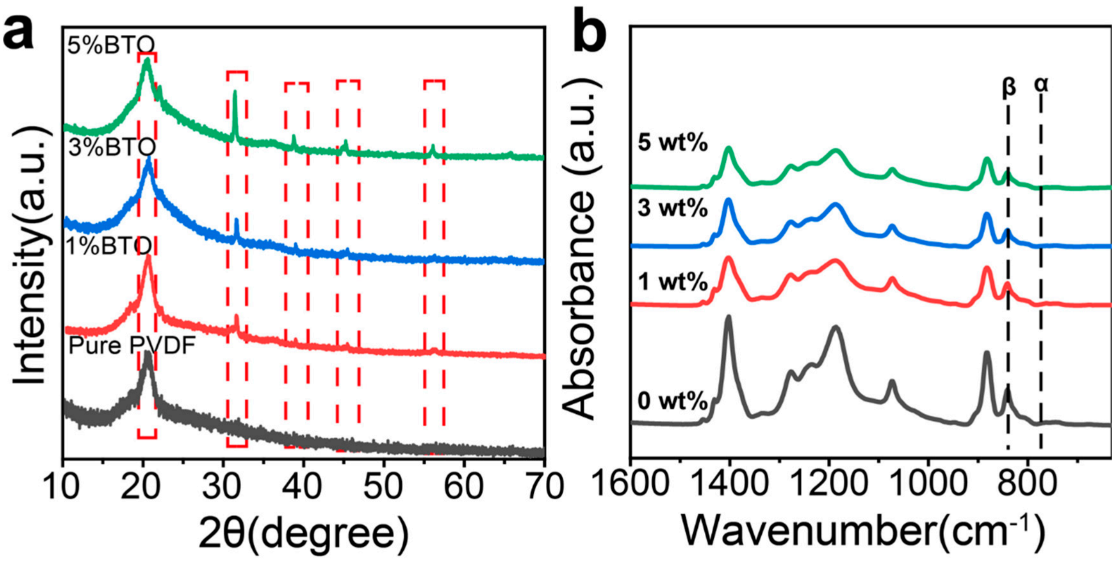

Figure S3. (a) X-ray diffraction (XRD) scattering pattern of the prepared electrospun fibers with various DET BTO mass fractions. (b) Fourier transform infrared (FTIR) spectra of the prepared electrospun fibers with various DET BTO mass fractions.

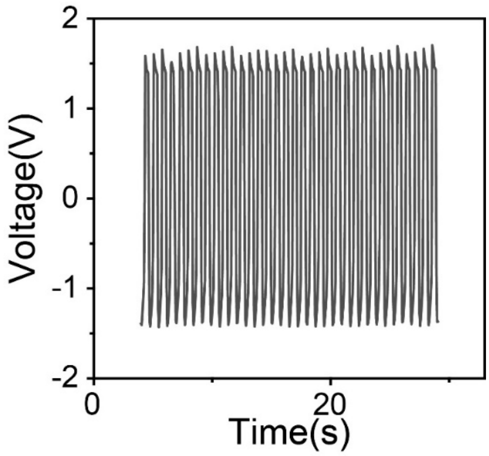

Figure S4. The output voltage of the prepared fibers with pure PVDF under a fixed stress of 18N.

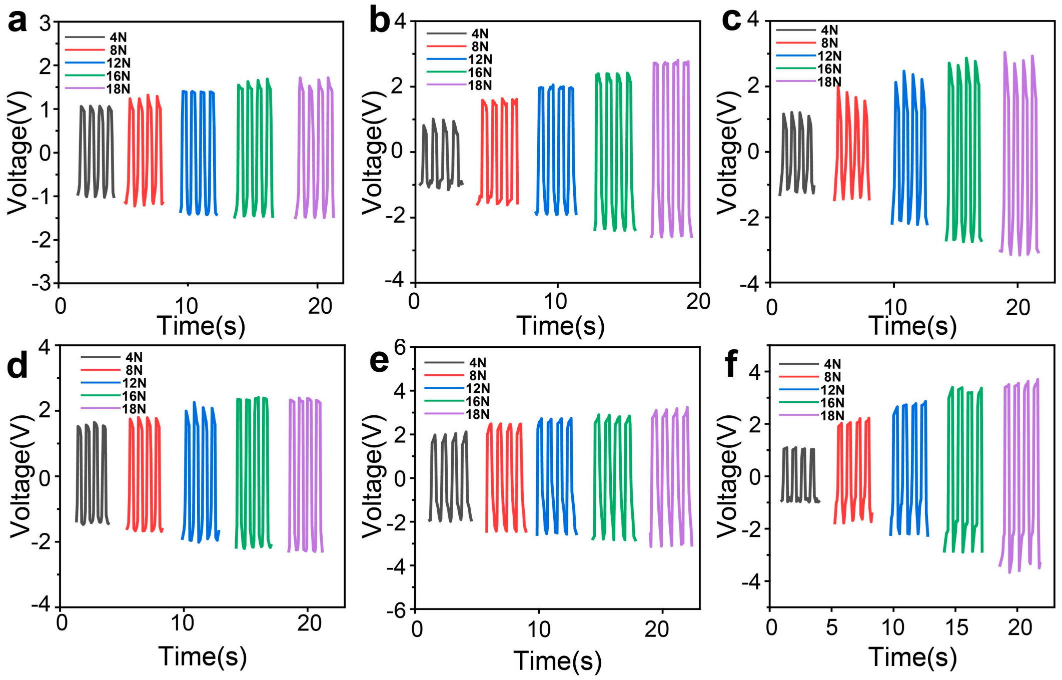

Figure S5. Output voltage of the prepared fibers under diverse external force. (a) pure PVDF, (b) 1wt% BTO/PVDF, (c) 3 wt% BTO/PVDF, (d) 5 wt% BTO/PVDF, (e) 1wt% DET BTO/PVDF, (f) 5 wt% DET BTO/PVDF.

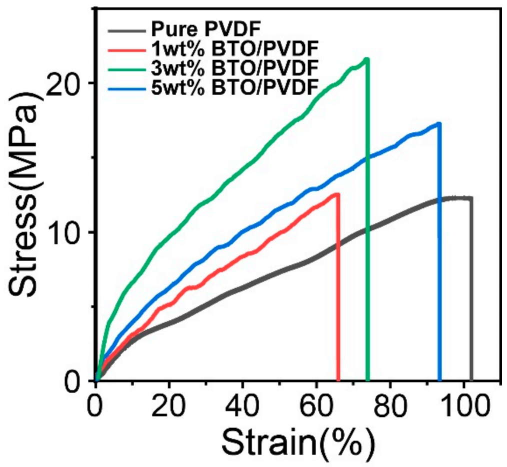

Figure S6. Stress-strain curves of the prepared fibers with various BTO mass fractions.

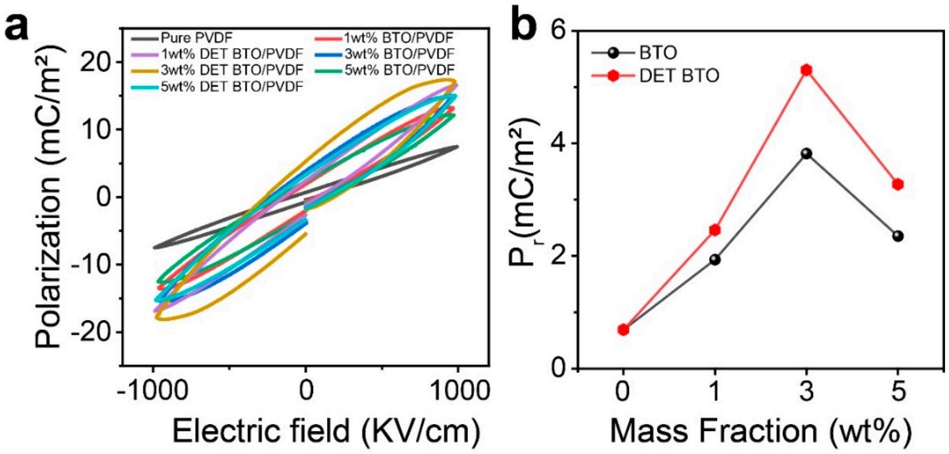

Figure S7. (a) Hysteresis loops of the prepared fibers with various BTO & DET BTO mass fractions at 1000 kV/cm, (b) The relationship between P_r values and the mass fractions of BTO & DET BTO at 1000 kV/cm.

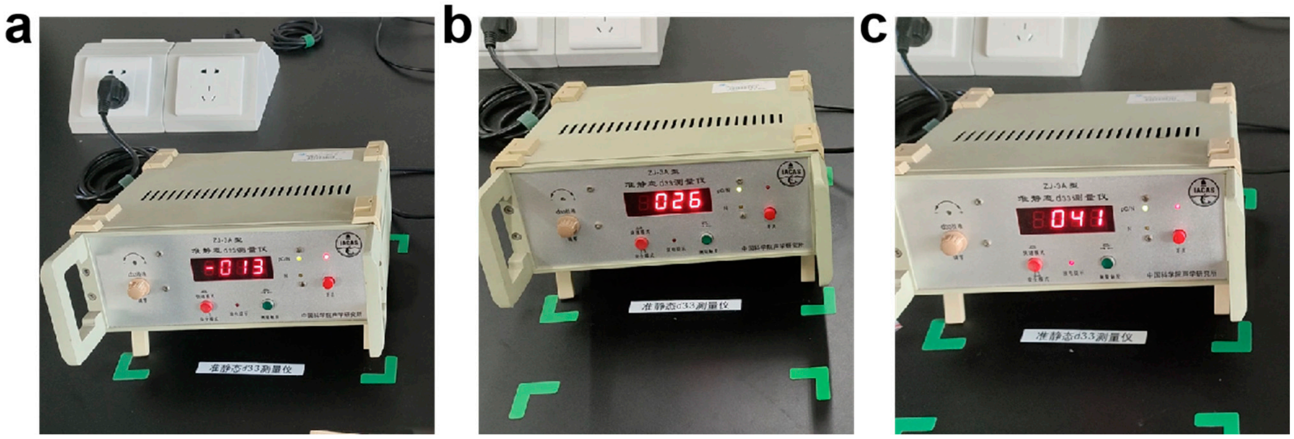

Figure S8. The d_{33} of (a) pure PVDF (b) 3 wt% BTO/PVDF (c) 3 wt% DET BTO/PVDF.

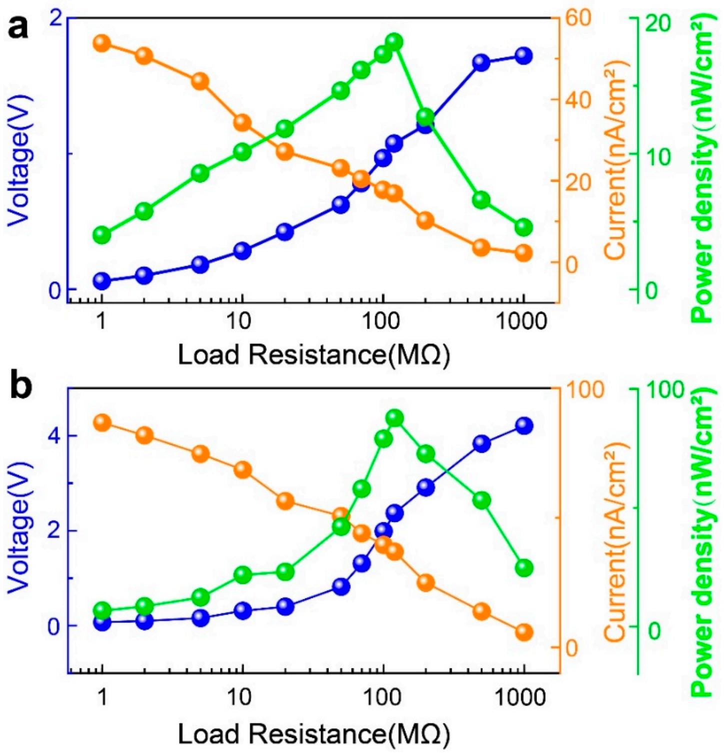

Figure S9. Voltage, current and power density of PEHs with various load resistance. (a) pure PVDF, (b) 3 wt% BTO/PVDF.

Table S1. Comparison between this work and previous studies

Materials	Structure	Peak-to-Peak Voltage	d_{33} (pC/N)	Ref.
P(VDF-TrFE)/PDA@BTO	nanofiber	6 V		[1]
PVDF/PDA@BTO	nanofiber	19.33 N/13 V	35.8	[2]
P(VDF-TrFE)/BTO	nanofiber	300 kPa/13.56 V	32.72	[3]
TOS-BTO/PVDF	Thin film	50 N/20 V	33.5	[4]
PVDF-*g*-MA/ PDA@BTO	Thin film	3.2 V	27.2	[5]
P(VDF-TrFE)/$BaTiO_3$ micropillars	Thin film	50 N/13.2 V	35.3	[6]
BTO@PDA/PVDF/BTO@PDA	Thin film	20 N/4.8 V	14.6	[7]
DET-BTO/PVDF	nanofiber	18 N/8.7 V Beat/27 V	40.3	This work

[1] X. Guan, B. Xu, J. Gong, Hierarchically architected polydopamine modified $BaTiO_3$@P(VDF-TrFE) nanocomposite fiber mats for flexible piezoelectric nanogenerators and self-powered sensors, Nano Energy 70 (2020) 104516.

[2] Y. Su, W. Li, L. Yuan, C. Chen, H. Pan, G. Xie, G. Conta, S. Ferrier, X. Zhao, G. Chen, H. Tai, Y. Jiang, J. Chen, Piezoelectric fiber composites with polydopamine interfacial layer for self-powered wearable biomonitoring, Nano Energy 89 (2021) 106321.

[3] S. Mirjalali, R. Bagherzadeh, A. Mahdavi Varposhti, M. Asadnia, S. Huang, W. Chang, S. Peng, C.-H. Wang, S. Wu, Enhanced Piezoelectricity of PVDF-TrFE Nanofibers by Intercalating with Electrosprayed $BaTiO_3$, ACS Appl. Mater. Interfaces 15 (2023) 41806–41816.

[4] H. Li, S. Lim, Screen Printing of Surface-Modified Barium Titanate/Polyvinylidene Fluoride Nanocomposites for High-Performance Flexible Piezoelectric Nanogenerators, Nanomaterials 12 (2022) 2910.

[5] L. Wang, T. Cheng, W. Lian, M. Zhang, B. Lu, B. Dong, K. Tan, C. Liu, C. Shen, Flexible layered cotton cellulose-based nanofibrous membranes for piezoelectric energy harvesting and self-powered sensing, Carbohydr. Polym. 275 (2022) 118740.

[6] X. Chen, X. Li, J. Shao, N. An, H. Tian, C. Wang, T. Han, L. Wang, B. Lu, High-Performance Piezoelectric Nanogenerators with Imprinted P(VDF-TrFE)/$BaTiO_3$ Nanocomposite Micropillars for Self-Powered Flexible Sensors, Small 13 (2017) 1604245.

[7] J. Le, F. Lv, J. Lin, Y. Wu, Z. Ren, Q. Zhang, S. Dong, J. Luo, J. Shi, R. Chen, Z. Hong, Y. Huang, Novel Sandwich-Structured Flexible Composite Films with Enhanced Piezoelectric Performance, ACS Appl. Mater. Interfaces 16 (2024) 1492–1501.
